# De­acetyl­cinobufalactam monohydrate

**DOI:** 10.1107/S1600536814010046

**Published:** 2014-05-10

**Authors:** Hong-Jin Tang, Xiao-Feng Yuan, Hai-Yan Tian, Li-Jun Ruan, Ren-Wang Jiang

**Affiliations:** a Guangdong Province Key Laboratory of Pharmacodynamic Constituents of Traditional Chinese Medicine and New Drugs Research, Institute of Traditional Chinese Medicine and Natural Products, Jinan University, Guangzhou 510632, People’s Republic of China

## Abstract

The title compound, C_24_H_33_NO_4_·H_2_O, the reaction product of de­acetyl­cinobufagin with ammonium acetate, consists of three cyclo­hexane rings (*A*, *B* and *C*), one five-membered ring (*D*), one six-membered lactone ring (*E*) and an epoxide ring (*F*). The stereochemistry of the ring junctures are *A*/*B cis*, *B*/*C trans*, *C*/*D cis* and *D*/*F cis*. Cyclo­hexane rings *A*, *B* and *C* have normal chair conformations. The five-membered ring *D* adopts an envelope conformation (with the C atom bearing the lactone ring as the flap) and the lactone ring *E* is planar. In the crystal, hy­droxy and water O—H⋯O and amine N—H⋯O hydrogen bonds involving carbonyl, hy­droxy and water O-atom acceptors link the mol­ecules into a three-dimensional network.

## Related literature   

For a previous isolation of de­acetyl­cinobufagin [cinobufagin systematic name: (3β,5β,15β,16β)-16-acet­oxy-3-hy­droxy-14,15-ep­oxy­bufa-20,22-dienolide] see: Li *et al.* (2007 ▶). For the biosynthesis of de­acetyl­cinobufagin, see: Zhan *et al.* (2003 ▶). For its pharmacological activity, see: Yu *et al.* (2008 ▶); Tian *et al.* (2013 ▶). For the stereochemistry of bufalin, see: Rohrer *et al.* (1982 ▶).
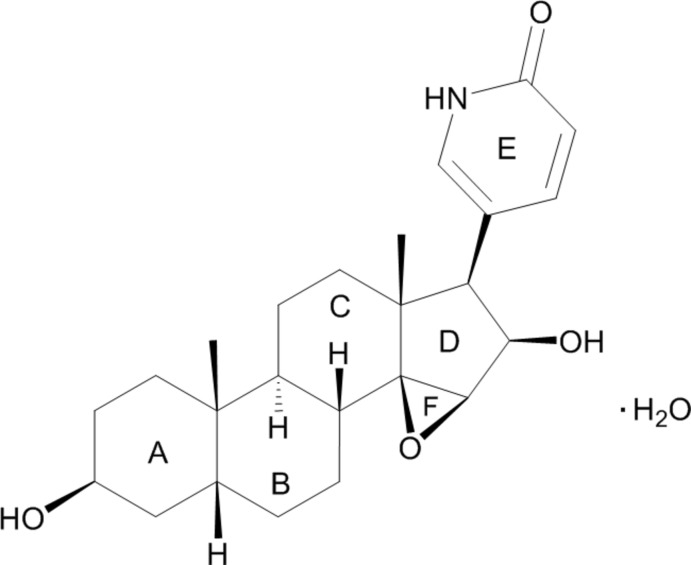


## Experimental   

### 

#### Crystal data  

C_24_H_33_NO_4_·H_2_O
*M* *_r_* = 417.53
Monoclinic, 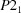

*a* = 8.0097 (2) Å
*b* = 12.1155 (4) Å
*c* = 11.3627 (3) Å
β = 95.077 (3)°
*V* = 1098.33 (5) Å^3^
*Z* = 2
Cu *K*α radiation
μ = 0.71 mm^−1^
*T* = 290 K
0.40 × 0.32 × 0.10 mm

#### Data collection  

Oxford Diffraction Gemini-S Ultra sapphire CCD diffractometer
Absorption correction: multi-scan (*CrysAlis PRO*; Agilent, 2011 ▶) *T*_min_ = 0.806, *T*_max_ = 1.0
3289 measured reflections
2396 independent reflections
2261 reflections with *I* > 2σ(*I*)
*R*_int_ = 0.018

#### Refinement  

*R*[*F* ^2^ > 2σ(*F* ^2^)] = 0.031
*wR*(*F* ^2^) = 0.081
*S* = 1.08
2396 reflections
280 parameters
1 restraint
H atoms treated by a mixture of independent and constrained refinement
Δρ_max_ = 0.14 e Å^−3^
Δρ_min_ = −0.13 e Å^−3^

### 

Data collection: *CrysAlis PRO* (Agilent, 2011 ▶); cell refinement: *CrysAlis PRO*; data reduction: *CrysAlis PRO*; program(s) used to solve structure: *SHELXS97* (Sheldrick, 2008 ▶); program(s) used to refine structure: *SHELXL97* (Sheldrick, 2008 ▶); molecular graphics: *XP* in *SHELXTL* (Sheldrick, 2008 ▶); software used to prepare material for publication: *SHELXTL*.

## Supplementary Material

Crystal structure: contains datablock(s) I, global. DOI: 10.1107/S1600536814010046/zs2298sup1.cif

Structure factors: contains datablock(s) I. DOI: 10.1107/S1600536814010046/zs2298Isup2.hkl

CCDC reference: 1000729

Additional supporting information:  crystallographic information; 3D view; checkCIF report

## Figures and Tables

**Table 1:** Hydrogen-bond geometry (Å, °)

*D*—H⋯*A*	*D*—H	H⋯*A*	*D*⋯*A*	*D*—H⋯*A*
O1*W*—H1*WA*⋯O4^i^	0.93 (4)	1.79 (4)	2.710 (3)	170 (4)
O1*W*—H1*WB*⋯O3	0.80 (5)	2.07 (5)	2.867 (3)	170 (4)
N1—H1*A*⋯O1^ii^	0.86	2.00	2.839 (3)	165
O1—H1*B*⋯O1*W* ^iii^	0.82	1.90	2.690 (3)	161
O3—H3*A*⋯O1^iv^	0.82	2.09	2.868 (2)	157

Symmetry codes: (i) 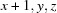; (ii) 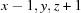; (iii) 
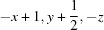
; (iv) 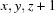.

**Fig. 1.**
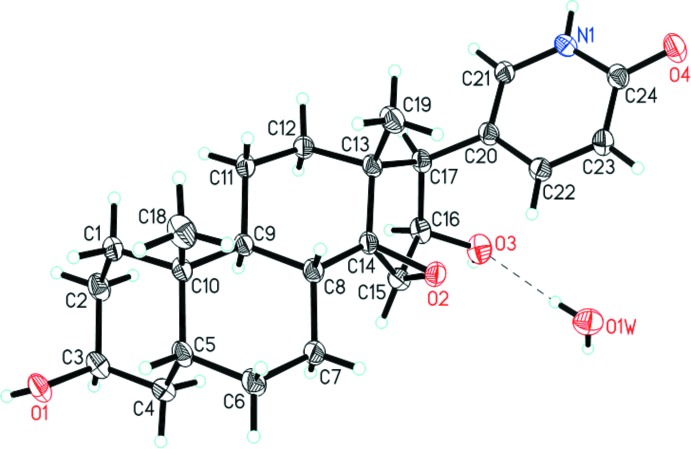
The molecular structure of the title compound showing atom the numbering scheme and 30% probability displacement ellipsoids. The inter-species hydrogen bond is shown as a dashed line.

**Fig. 2.**
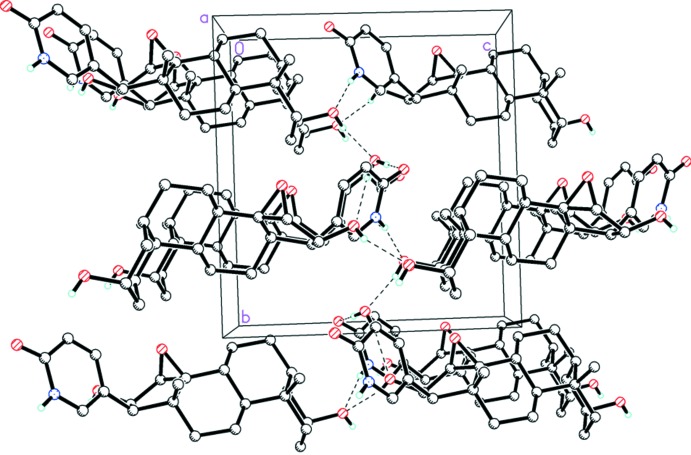
The packing diagram showing the intermolecular O—H···O and N—H···O hydrogen bonds which are represented by dashed lines. Selected H-atoms highlighting the hydrogen bonding are shown.

## supplementary crystallographic information

### 1. Comment

Deacetylcinobufagin is a natural cardiactonic steroid which has been isolated from the skin of the toad (Li *et al.*, 2007) and has also been biosynthesized by microbial transformation of cinobufagin (Zhan *et al.*, 2003). Compounds of this type have shown strong cytotoxic effects against a wide range of cancer cells (Yu *et al.*, 2008). However they also possess cardiac toxicity due to the inhibition of sodium-potassium ATPase (Tian *et al.*, 2013). Thus structural modification of the pharmacological profile of the molecule was warranted. Recently we treated deacetylcinobufagin (isolated in our laboratory) with ammonium acetate, and a new hydrated derivative, C_24_H_33_O_4_N . H_2_O, the title compound, named deacetylcinobufalactam, was obtained after recrystallization from methanol at room temperature. We report herein the crystal structure of this compound.

The molecule of the title compound (Fig. 1) consists of three cyclohexane rings (*A*, *B* and *C*), one five-membered ring (*D*), one six-membered lactam ring (*E*) and an epoxide ring (*F*). The stereochemistry of the ring juncture is *A/B**cis*, *B/C**trans*, *C/D**cis* and *D/F**cis*. The cyclohexane rings *A*, *B* and *C* have normal chair conformations. The five-membered ring (*D* adopts an envelope conformation with C17 displaced by -0.381 (3) Å from the mean plane of the remaining four atoms (C13, C14, C15 and C16). The lactam ring (*E*) and the epoxide ring (*F*) are planar and roughly perpendicular to each other with a dihedral angle of 96.6 (4)°. The absolute configuration determined for bufalin (Rohrer *et al.*, 1982), a similar cardiactonic steroid, was invoked, giving the assignments of the 10 chiral centres in the title molecule as shown in Fig. 1.

In the crystal, intermolecular hydroxyl and water O—H···O hydrogen bonds to hydroxyl, carbonyl and water O-atom acceptors and a hetero-amine N—H···O_hydroxyl_ hydrogen bond (Table 1) link the molecules into a three-dimensional network structure (Figure 2).

### 2. Experimental

Deacetylcinobufagin (40.0 mg) was dissolved in DMF, then ammonium acetate (38.5 mg) was added under nitrogen protection. The mixture was stirred for three hours at 100 °C. After completion of the reaction, the mixture was poured into water and extracted with ethyl acetate. The ethyl acetate extract was washed with water to remove the solvent DMF and the excess ammonium acetate and condensed by rotary evaporation under reduced pressure. The residue was recrystallized in methanol at room temperature to afford colorless crystals (28.6 mg, yield 71.7%).

### 3. Refinement

The C-bound H atoms were positioned geometrically and were included in the refinement in the riding-model approximation, with C—H = 0.96 Å (CH_3_) and *U*_iso_(H) = 1.5*U*_eq_(C); 0.97 Å (CH_2_) and *U*_iso_(H) = 1.2*U*_eq_(C); 0.98 Å (CH) and *U*_iso_(H) = 1.2*U*_eq_(C); 0.93 Å (aryl H) and *U*_iso_(H)= 1.2*U*_eq_(C); O—H = 0.82 Å and *U*_iso_(H) = 1.5*U*_eq_(O). The Friedel pair coverage for the collection is low. It may be due to an inadequate collection strategy. Recollection of diffraction data was not thought to be necessary since the absolute configuration can be unambiguously assigned with reference to the known configuration of the closely related compound bufalin (Rohrer *et al.*, 1982) [(C3*S*,C5*R*, C8*R*,C9*S*,C10*S*,C13*R*,C14*S*,C15*R*, C16*R*,C17*R*) for the 10 chiral centres in the title compound using the arbitrarily named atoms employed]. The Flack parameter was refined to 0.0 (3) for 571 Friedel pairs. There are 32 reflections missing between θ(min) and θ(max), which might be also due to the inadequate collection strategy, and adjustment of the orientation to tilt the crystal axis might be helpful for collecting a complete set of diffraction data. In addition, both hydrogen atoms on the water molecule are involved in hydrogen bonding. The O—H bond distances are significantly different from the ideal bond length so these two hydrogen atoms were refined freely. The highest residual electron density was 0.142 eÅ^3^ and has no particular structural significance.

### Crystal data

**Table:**

C_24_H_33_NO_4_·H_2_O	*F*(000) = 452
*M**_r_* = 417.53	*D*_x_ = 1.263 Mg m^−^^3^
Monoclinic, *P*2_1_	Cu *K*α radiation, λ = 1.54184 Å
Hall symbol: P 2yb	Cell parameters from 2374 reflections
*a* = 8.0097 (2) Å	θ = 3.9–62.8°
*b* = 12.1155 (4) Å	µ = 0.71 mm^−^^1^
*c* = 11.3627 (3) Å	*T* = 290 K
β = 95.077 (3)°	Plate, colorless
*V* = 1098.33 (5) Å^3^	0.40 × 0.32 × 0.10 mm
*Z* = 2	

### Data collection

**Table:**

Oxford Diffraction Gemini-S Ultra sapphire CCD diffractometer	2396 independent reflections
Radiation source: fine-focus sealed tube	2261 reflections with *I* > 2σ(*I*)
Graphite monochromator	*R*_int_ = 0.018
ω scans	θ_max_ = 62.8°, θ_min_ = 3.9°
Absorption correction: multi-scan (*CrysAlis PRO*; Agilent, 2011)	*h* = −9→8
*T*_min_ = 0.806, *T*_max_ = 1.0	*k* = −8→13
3289 measured reflections	*l* = −11→13

### Refinement

**Table:**

Refinement on *F*^2^	Primary atom site location: structure-invariant direct methods
Least-squares matrix: full	Secondary atom site location: difference Fourier map
*R*[*F*^2^ > 2σ(*F*^2^)] = 0.031	Hydrogen site location: inferred from neighbouring sites
*wR*(*F*^2^) = 0.081	H atoms treated by a mixture of independent and constrained refinement
*S* = 1.08	*w* = 1/[σ^2^(*F*_o_^2^) + (0.0421*P*)^2^ + 0.128*P*] where *P* = (*F*_o_^2^ + 2*F*_c_^2^)/3
2396 reflections	(Δ/σ)_max_ < 0.001
280 parameters	Δρ_max_ = 0.14 e Å^−^^3^
1 restraint	Δρ_min_ = −0.13 e Å^−^^3^

### Special details

**Table:**

Geometry. All e.s.d.'s (except the e.s.d. in the dihedral angle between two l.s. planes) are estimated using the full covariance matrix. The cell e.s.d.'s are taken into account individually in the estimation of e.s.d.'s in distances, angles and torsion angles; correlations between e.s.d.'s in cell parameters are only used when they are defined by crystal symmetry. An approximate (isotropic) treatment of cell e.s.d.'s is used for estimating e.s.d.'s involving l.s. planes.
Refinement. Refinement of *F*^2^ against ALL reflections. The weighted *R*-factor *wR* and goodness of fit *S* are based on *F*^2^, conventional *R*-factors *R* are based on *F*, with *F* set to zero for negative *F*^2^. The threshold expression of *F*^2^ > σ(*F*^2^) is used only for calculating *R*-factors(gt) *etc*. and is not relevant to the choice of reflections for refinement. *R*-factors based on *F*^2^ are statistically about twice as large as those based on *F*, and *R*- factors based on ALL data will be even larger.

### Fractional atomic coordinates and isotropic or equivalent isotropic displacement parameters (Å^2^)

**Table:**

	*x*	*y*	*z*	*U*_iso_*/*U*_eq_	
N1	−0.2500 (2)	0.63824 (18)	0.52081 (16)	0.0396 (5)	
H1A	−0.3296	0.6720	0.5520	0.048*	
O1	0.5226 (2)	0.78557 (15)	−0.38275 (14)	0.0450 (4)	
H1B	0.5728	0.8339	−0.4159	0.067*	
O2	0.2223 (2)	0.53707 (14)	0.21889 (13)	0.0404 (4)	
O3	0.3082 (2)	0.66560 (16)	0.44674 (14)	0.0506 (5)	
H3A	0.3830	0.7042	0.4788	0.076*	
O4	−0.3079 (2)	0.48508 (18)	0.62396 (15)	0.0529 (5)	
C1	0.2137 (3)	0.8092 (2)	−0.2603 (2)	0.0375 (5)	
H1C	0.2009	0.7931	−0.3442	0.045*	
H1D	0.1184	0.8536	−0.2424	0.045*	
C2	0.3714 (3)	0.8770 (2)	−0.2341 (2)	0.0430 (6)	
H2A	0.3799	0.9003	−0.1521	0.052*	
H2B	0.3653	0.9426	−0.2832	0.052*	
C3	0.5255 (3)	0.8109 (2)	−0.2575 (2)	0.0410 (6)	
H3B	0.6263	0.8537	−0.2327	0.049*	
C4	0.5291 (3)	0.7026 (2)	−0.1897 (2)	0.0399 (6)	
H4A	0.6233	0.6591	−0.2113	0.048*	
H4B	0.5473	0.7185	−0.1058	0.048*	
C5	0.3701 (3)	0.6345 (2)	−0.21181 (18)	0.0347 (5)	
H5A	0.3617	0.6135	−0.2954	0.042*	
C6	0.3824 (4)	0.5274 (2)	−0.1411 (2)	0.0465 (6)	
H6A	0.4896	0.4928	−0.1504	0.056*	
H6B	0.2951	0.4773	−0.1727	0.056*	
C7	0.3649 (3)	0.5455 (2)	−0.0095 (2)	0.0449 (6)	
H7A	0.3630	0.4745	0.0299	0.054*	
H7B	0.4614	0.5862	0.0251	0.054*	
C8	0.2052 (3)	0.6091 (2)	0.01045 (19)	0.0343 (5)	
H8A	0.1104	0.5640	−0.0218	0.041*	
C9	0.1961 (3)	0.71946 (19)	−0.05767 (19)	0.0305 (5)	
H9A	0.2951	0.7624	−0.0287	0.037*	
C10	0.2087 (3)	0.6997 (2)	−0.19199 (18)	0.0328 (5)	
C11	0.0424 (3)	0.7867 (2)	−0.0302 (2)	0.0420 (6)	
H11A	0.0466	0.8585	−0.0675	0.050*	
H11B	−0.0581	0.7494	−0.0634	0.050*	
C12	0.0323 (3)	0.8024 (2)	0.1027 (2)	0.0405 (6)	
H12A	−0.0680	0.8441	0.1151	0.049*	
H12B	0.1281	0.8452	0.1345	0.049*	
C13	0.0287 (3)	0.69225 (19)	0.17083 (18)	0.0324 (5)	
C14	0.1819 (3)	0.62930 (19)	0.13928 (18)	0.0320 (5)	
C15	0.3140 (3)	0.6388 (2)	0.2355 (2)	0.0392 (5)	
H15A	0.4314	0.6419	0.2177	0.047*	
C16	0.2557 (3)	0.7099 (2)	0.33232 (19)	0.0387 (6)	
H16A	0.3031	0.7840	0.3260	0.046*	
C17	0.0623 (3)	0.71678 (19)	0.30680 (18)	0.0336 (5)	
H17A	0.0313	0.7941	0.3181	0.040*	
C18	0.0561 (3)	0.6373 (3)	−0.2475 (2)	0.0516 (7)	
H18A	0.0675	0.6258	−0.3300	0.077*	
H18B	−0.0433	0.6797	−0.2385	0.077*	
H18C	0.0479	0.5673	−0.2090	0.077*	
C19	−0.1342 (3)	0.6303 (3)	0.1395 (2)	0.0452 (6)	
H19A	−0.1484	0.6179	0.0558	0.068*	
H19B	−0.2264	0.6732	0.1630	0.068*	
H19C	−0.1307	0.5607	0.1800	0.068*	
C20	−0.0349 (3)	0.6490 (2)	0.38948 (18)	0.0329 (5)	
C21	−0.1599 (3)	0.6964 (2)	0.44449 (19)	0.0358 (5)	
H21A	−0.1854	0.7703	0.4301	0.043*	
C22	−0.0037 (3)	0.5362 (2)	0.41596 (18)	0.0349 (5)	
H22A	0.0805	0.4998	0.3800	0.042*	
C23	−0.0930 (3)	0.4799 (2)	0.49229 (19)	0.0375 (5)	
H23A	−0.0689	0.4058	0.5068	0.045*	
C24	−0.2220 (3)	0.5307 (2)	0.55044 (19)	0.0379 (6)	
O1W	0.3771 (3)	0.4443 (2)	0.5263 (2)	0.0555 (5)	
H1WA	0.480 (5)	0.462 (3)	0.567 (3)	0.092 (13)*	
H1WB	0.355 (5)	0.503 (4)	0.496 (3)	0.084 (14)*	

### Atomic displacement parameters (Å^2^)

**Table:**

	*U*^11^	*U*^22^	*U*^33^	*U*^12^	*U*^13^	*U*^23^
N1	0.0374 (10)	0.0467 (13)	0.0365 (10)	0.0049 (9)	0.0131 (8)	0.0015 (10)
O1	0.0544 (10)	0.0426 (11)	0.0413 (9)	−0.0053 (8)	0.0232 (8)	0.0012 (8)
O2	0.0541 (9)	0.0351 (9)	0.0335 (8)	0.0123 (8)	0.0119 (7)	0.0039 (8)
O3	0.0563 (10)	0.0580 (12)	0.0357 (9)	−0.0091 (9)	−0.0054 (7)	−0.0029 (9)
O4	0.0498 (10)	0.0619 (12)	0.0490 (10)	−0.0076 (9)	0.0154 (8)	0.0124 (10)
C1	0.0428 (12)	0.0421 (14)	0.0288 (12)	0.0056 (11)	0.0091 (9)	0.0046 (11)
C2	0.0593 (15)	0.0318 (13)	0.0403 (13)	−0.0022 (12)	0.0179 (11)	−0.0008 (11)
C3	0.0437 (13)	0.0431 (14)	0.0380 (13)	−0.0094 (11)	0.0139 (10)	−0.0045 (12)
C4	0.0373 (12)	0.0458 (15)	0.0379 (12)	0.0043 (11)	0.0105 (10)	0.0002 (12)
C5	0.0467 (12)	0.0323 (13)	0.0269 (10)	−0.0007 (11)	0.0132 (9)	−0.0032 (10)
C6	0.0635 (15)	0.0373 (15)	0.0422 (13)	0.0084 (12)	0.0240 (11)	0.0007 (12)
C7	0.0616 (15)	0.0407 (14)	0.0353 (12)	0.0193 (13)	0.0209 (11)	0.0068 (12)
C8	0.0421 (12)	0.0317 (13)	0.0304 (11)	0.0025 (10)	0.0100 (9)	0.0012 (10)
C9	0.0351 (11)	0.0301 (12)	0.0274 (11)	0.0008 (9)	0.0084 (8)	0.0013 (10)
C10	0.0342 (11)	0.0378 (13)	0.0273 (11)	−0.0027 (10)	0.0067 (8)	0.0021 (11)
C11	0.0512 (14)	0.0439 (16)	0.0330 (13)	0.0135 (12)	0.0156 (10)	0.0118 (11)
C12	0.0526 (14)	0.0336 (13)	0.0377 (13)	0.0144 (12)	0.0181 (11)	0.0054 (11)
C13	0.0390 (12)	0.0310 (12)	0.0287 (11)	0.0024 (10)	0.0105 (9)	0.0000 (10)
C14	0.0406 (11)	0.0262 (12)	0.0305 (11)	0.0025 (10)	0.0116 (9)	0.0036 (10)
C15	0.0346 (11)	0.0459 (15)	0.0379 (12)	0.0011 (11)	0.0080 (9)	0.0021 (12)
C16	0.0462 (13)	0.0370 (14)	0.0335 (12)	−0.0076 (11)	0.0065 (10)	−0.0030 (11)
C17	0.0444 (12)	0.0273 (12)	0.0306 (12)	0.0012 (10)	0.0117 (9)	−0.0019 (10)
C18	0.0508 (14)	0.0668 (19)	0.0370 (13)	−0.0149 (14)	0.0033 (10)	−0.0092 (14)
C19	0.0417 (12)	0.0595 (17)	0.0353 (12)	−0.0028 (13)	0.0082 (10)	−0.0001 (13)
C20	0.0382 (11)	0.0357 (13)	0.0258 (10)	0.0009 (10)	0.0076 (9)	−0.0025 (10)
C21	0.0391 (12)	0.0361 (13)	0.0332 (11)	0.0050 (11)	0.0088 (9)	0.0038 (11)
C22	0.0401 (12)	0.0357 (13)	0.0295 (11)	0.0019 (11)	0.0063 (9)	−0.0037 (11)
C23	0.0468 (12)	0.0336 (13)	0.0326 (11)	−0.0016 (11)	0.0058 (10)	0.0011 (11)
C24	0.0361 (12)	0.0457 (15)	0.0316 (11)	−0.0050 (11)	0.0010 (9)	0.0034 (12)
O1W	0.0558 (13)	0.0493 (13)	0.0618 (13)	0.0030 (10)	0.0073 (10)	−0.0124 (12)

### Geometric parameters (Å, º)

**Table:**

O1—C3	1.454 (3)	C20—C22	1.417 (3)
O2—C14	1.456 (3)	C20—C21	1.354 (3)
O2—C15	1.439 (3)	C22—C23	1.356 (3)
O3—C16	1.435 (3)	C23—C24	1.416 (3)
O4—C24	1.256 (3)	C1—H1C	0.9700
O1—H1B	0.8200	C1—H1D	0.9700
O3—H3A	0.8200	C2—H2B	0.9700
O1W—H1WB	0.80 (5)	C2—H2A	0.9700
O1W—H1WA	0.93 (4)	C3—H3B	0.9800
N1—C24	1.359 (3)	C4—H4A	0.9700
N1—C21	1.371 (3)	C4—H4B	0.9700
N1—H1A	0.8600	C5—H5A	0.9800
C1—C2	1.514 (3)	C6—H6B	0.9700
C1—C10	1.539 (3)	C6—H6A	0.9700
C2—C3	1.515 (3)	C7—H7B	0.9700
C3—C4	1.521 (3)	C7—H7A	0.9700
C4—C5	1.520 (3)	C8—H8A	0.9800
C5—C10	1.548 (3)	C9—H9A	0.9800
C5—C6	1.525 (3)	C11—H11A	0.9700
C6—C7	1.530 (3)	C11—H11B	0.9700
C7—C8	1.527 (3)	C12—H12A	0.9700
C8—C14	1.512 (3)	C12—H12B	0.9700
C8—C9	1.543 (3)	C15—H15A	0.9800
C9—C10	1.557 (3)	C16—H16A	0.9800
C9—C11	1.531 (3)	C17—H17A	0.9800
C10—C18	1.525 (4)	C18—H18B	0.9600
C11—C12	1.531 (3)	C18—H18A	0.9600
C12—C13	1.544 (3)	C18—H18C	0.9600
C13—C19	1.520 (4)	C19—H19B	0.9600
C13—C14	1.515 (3)	C19—H19C	0.9600
C13—C17	1.573 (3)	C19—H19A	0.9600
C14—C15	1.457 (3)	C21—H21A	0.9300
C15—C16	1.504 (3)	C22—H22A	0.9300
C16—C17	1.553 (3)	C23—H23A	0.9300
C17—C20	1.514 (3)		
			
C14—O2—C15	60.45 (15)	C3—C2—H2A	109.00
C3—O1—H1B	109.00	C1—C2—H2A	109.00
C16—O3—H3A	109.00	H2A—C2—H2B	108.00
H1WA—O1W—H1WB	99 (4)	C3—C2—H2B	109.00
C21—N1—C24	124.38 (19)	O1—C3—H3B	110.00
C21—N1—H1A	118.00	C4—C3—H3B	109.00
C24—N1—H1A	118.00	C2—C3—H3B	109.00
C2—C1—C10	115.27 (19)	C3—C4—H4B	109.00
C1—C2—C3	110.9 (2)	C5—C4—H4A	109.00
O1—C3—C4	108.16 (19)	C5—C4—H4B	109.00
C2—C3—C4	110.28 (19)	H4A—C4—H4B	108.00
O1—C3—C2	109.92 (18)	C3—C4—H4A	109.00
C3—C4—C5	114.00 (19)	C6—C5—H5A	107.00
C4—C5—C10	113.4 (2)	C10—C5—H5A	107.00
C6—C5—C10	111.9 (2)	C4—C5—H5A	107.00
C4—C5—C6	111.2 (2)	C5—C6—H6B	109.00
C5—C6—C7	112.69 (19)	C7—C6—H6A	109.00
C6—C7—C8	111.6 (2)	C7—C6—H6B	109.00
C9—C8—C14	109.93 (19)	H6A—C6—H6B	108.00
C7—C8—C14	113.71 (19)	C5—C6—H6A	109.00
C7—C8—C9	111.45 (19)	C6—C7—H7A	109.00
C8—C9—C11	111.21 (19)	C6—C7—H7B	109.00
C8—C9—C10	110.74 (18)	C8—C7—H7B	109.00
C10—C9—C11	113.87 (19)	H7A—C7—H7B	108.00
C5—C10—C18	109.6 (2)	C8—C7—H7A	109.00
C1—C10—C18	105.9 (2)	C7—C8—H8A	107.00
C1—C10—C5	107.88 (19)	C9—C8—H8A	107.00
C9—C10—C18	111.25 (19)	C14—C8—H8A	107.00
C1—C10—C9	111.63 (19)	C8—C9—H9A	107.00
C5—C10—C9	110.39 (18)	C10—C9—H9A	107.00
C9—C11—C12	112.38 (19)	C11—C9—H9A	107.00
C11—C12—C13	113.08 (19)	C9—C11—H11B	109.00
C14—C13—C17	104.82 (18)	C12—C11—H11A	109.00
C12—C13—C14	105.37 (18)	C9—C11—H11A	109.00
C14—C13—C19	113.1 (2)	H11A—C11—H11B	108.00
C17—C13—C19	113.08 (18)	C12—C11—H11B	109.00
C12—C13—C17	108.66 (18)	C11—C12—H12A	109.00
C12—C13—C19	111.3 (2)	C13—C12—H12A	109.00
O2—C14—C8	115.95 (19)	C13—C12—H12B	109.00
O2—C14—C15	59.19 (14)	H12A—C12—H12B	108.00
C8—C14—C13	118.86 (19)	C11—C12—H12B	109.00
O2—C14—C13	112.31 (17)	O2—C15—H15A	120.00
C13—C14—C15	109.41 (18)	C14—C15—H15A	120.00
C8—C14—C15	126.6 (2)	C16—C15—H15A	120.00
O2—C15—C16	113.50 (19)	O3—C16—H16A	109.00
O2—C15—C14	60.36 (14)	C15—C16—H16A	109.00
C14—C15—C16	110.0 (2)	C17—C16—H16A	109.00
C15—C16—C17	105.27 (18)	C16—C17—H17A	107.00
O3—C16—C17	113.38 (18)	C20—C17—H17A	107.00
O3—C16—C15	111.32 (19)	C13—C17—H17A	107.00
C16—C17—C20	114.47 (18)	C10—C18—H18B	109.00
C13—C17—C20	117.02 (19)	C10—C18—H18C	110.00
C13—C17—C16	104.74 (18)	C10—C18—H18A	109.00
C17—C20—C21	119.9 (2)	H18A—C18—H18C	109.00
C21—C20—C22	115.8 (2)	H18B—C18—H18C	109.00
C17—C20—C22	124.4 (2)	H18A—C18—H18B	109.00
N1—C21—C20	121.7 (2)	C13—C19—H19A	109.00
C20—C22—C23	121.9 (2)	C13—C19—H19B	109.00
C22—C23—C24	121.9 (2)	H19A—C19—H19B	109.00
O4—C24—C23	125.8 (2)	H19A—C19—H19C	109.00
O4—C24—N1	119.9 (2)	C13—C19—H19C	109.00
N1—C24—C23	114.4 (2)	H19B—C19—H19C	109.00
C2—C1—H1C	108.00	C20—C21—H21A	119.00
C10—C1—H1C	108.00	N1—C21—H21A	119.00
C10—C1—H1D	108.00	C20—C22—H22A	119.00
C2—C1—H1D	108.00	C23—C22—H22A	119.00
H1C—C1—H1D	108.00	C22—C23—H23A	119.00
C1—C2—H2B	109.00	C24—C23—H23A	119.00
			
C15—O2—C14—C8	118.8 (2)	C10—C9—C11—C12	178.82 (19)
C15—O2—C14—C13	−100.0 (2)	C9—C11—C12—C13	−57.6 (3)
C14—O2—C15—C16	100.5 (2)	C11—C12—C13—C14	54.6 (2)
C24—N1—C21—C20	1.4 (3)	C11—C12—C13—C17	166.44 (19)
C21—N1—C24—O4	177.9 (2)	C11—C12—C13—C19	−68.4 (3)
C21—N1—C24—C23	−2.1 (3)	C12—C13—C14—O2	164.85 (17)
C10—C1—C2—C3	56.9 (3)	C12—C13—C14—C8	−55.1 (3)
C2—C1—C10—C5	−53.0 (2)	C12—C13—C14—C15	101.1 (2)
C2—C1—C10—C9	68.5 (3)	C17—C13—C14—O2	50.3 (2)
C2—C1—C10—C18	−170.3 (2)	C17—C13—C14—C8	−169.7 (2)
C1—C2—C3—O1	65.1 (2)	C17—C13—C14—C15	−13.5 (2)
C1—C2—C3—C4	−54.1 (2)	C19—C13—C14—O2	−73.4 (2)
O1—C3—C4—C5	−66.7 (2)	C19—C13—C14—C8	66.7 (3)
C2—C3—C4—C5	53.6 (3)	C19—C13—C14—C15	−137.1 (2)
C3—C4—C5—C6	−179.94 (18)	C12—C13—C17—C16	−89.8 (2)
C3—C4—C5—C10	−52.8 (2)	C12—C13—C17—C20	142.3 (2)
C4—C5—C6—C7	74.5 (3)	C14—C13—C17—C16	22.5 (2)
C10—C5—C6—C7	−53.5 (3)	C14—C13—C17—C20	−105.5 (2)
C4—C5—C10—C1	49.7 (2)	C19—C13—C17—C16	146.2 (2)
C4—C5—C10—C9	−72.6 (2)	C19—C13—C17—C20	18.2 (3)
C4—C5—C10—C18	164.56 (19)	O2—C14—C15—C16	−106.3 (2)
C6—C5—C10—C1	176.39 (18)	C8—C14—C15—O2	−101.1 (2)
C6—C5—C10—C9	54.2 (3)	C8—C14—C15—C16	152.6 (2)
C6—C5—C10—C18	−68.7 (2)	C13—C14—C15—O2	104.98 (19)
C5—C6—C7—C8	53.6 (3)	C13—C14—C15—C16	−1.3 (3)
C6—C7—C8—C9	−55.0 (3)	O2—C15—C16—O3	73.6 (2)
C6—C7—C8—C14	−180.0 (2)	O2—C15—C16—C17	−49.7 (2)
C7—C8—C9—C10	56.5 (2)	C14—C15—C16—O3	139.0 (2)
C7—C8—C9—C11	−175.82 (18)	C14—C15—C16—C17	15.8 (2)
C14—C8—C9—C10	−176.48 (19)	O3—C16—C17—C13	−145.25 (19)
C14—C8—C9—C11	−48.8 (3)	O3—C16—C17—C20	−15.8 (3)
C7—C8—C14—O2	−41.8 (3)	C15—C16—C17—C13	−23.3 (2)
C7—C8—C14—C13	179.6 (2)	C15—C16—C17—C20	106.2 (2)
C7—C8—C14—C15	27.8 (3)	C13—C17—C20—C21	−107.9 (2)
C9—C8—C14—O2	−167.51 (19)	C13—C17—C20—C22	72.7 (3)
C9—C8—C14—C13	53.9 (3)	C16—C17—C20—C21	129.0 (2)
C9—C8—C14—C15	−97.9 (3)	C16—C17—C20—C22	−50.4 (3)
C8—C9—C10—C1	−175.57 (19)	C17—C20—C21—N1	−179.4 (2)
C8—C9—C10—C5	−55.6 (3)	C22—C20—C21—N1	0.0 (3)
C8—C9—C10—C18	66.3 (3)	C17—C20—C22—C23	178.9 (2)
C11—C9—C10—C1	58.2 (3)	C21—C20—C22—C23	−0.5 (3)
C11—C9—C10—C5	178.21 (19)	C20—C22—C23—C24	−0.3 (4)
C11—C9—C10—C18	−59.9 (3)	C22—C23—C24—O4	−178.5 (2)
C8—C9—C11—C12	52.9 (3)	C22—C23—C24—N1	1.5 (3)

### Hydrogen-bond geometry (Å, º)

**Table:**

*D*—H···*A*	*D*—H	H···*A*	*D*···*A*	*D*—H···*A*
O1*W*—H1*WA*···O4^i^	0.93 (4)	1.79 (4)	2.710 (3)	170 (4)
O1*W*—H1*WB*···O3	0.80 (5)	2.07 (5)	2.867 (3)	170 (4)
N1—H1*A*···O1^ii^	0.86	2.00	2.839 (3)	165
O1—H1*B*···O1*W*^iii^	0.82	1.90	2.690 (3)	161
O3—H3*A*···O1^iv^	0.82	2.09	2.868 (2)	157

Symmetry codes: (i) *x*+1, *y*, *z*; (ii) *x*−1, *y*, *z*+1; (iii) −*x*+1, *y*+1/2, −*z*; (iv) *x*, *y*, *z*+1.
